# A hybrid approach for real-time hand tracking using fiducial markers and inertial sensors^✰^

**DOI:** 10.1016/j.mex.2025.103609

**Published:** 2025-09-08

**Authors:** Ranjeet Bidwe, Shubhangi Deokar, Yash Parkhi, Tanisha Vyas, Nimita Jestin, Utkarsh Kumar, Satviki Budhia, Armaan Jeswani

**Affiliations:** a Department of Computer Science and Engineering, Symbiosis Institute of Technology, Pune Campus, Symbiosis International (Deemed University), Pune 412115, India; b Department of Robotics and Automation, Symbiosis Institute of Technology, Pune Campus, Symbiosis International (Deemed University), Pune 412115, India

**Keywords:** Hand tracking, Fiducial markers, Computer vision, Capacitive touch sensors, OpenCV, ArUco markers, Inertial measurement unit (IMU), Bluetooth Low Energy (BLE)

## Abstract

This paper presents a cost-effective hybrid hand-tracking technique that integrates fiducial marker detection, capacitive touch sensing, and inertial measurement for real-time gesture recognition in immersive environments. The system is implemented on lightweight hardware comprising a Raspberry Pi Zero 2 W and an ESP32, with OpenCV’s ArUco marker detection enabling 3D hand pose estimation, capacitive sensors supporting finger-state recognition, and an Inertial Measurement Unit (IMU) providing orientation tracking. Optimizations such as exposure adjustment and region-of-interest processing ensure robust marker detection under variable illumination, while sensor data is transmitted via Bluetooth Low Energy (BLE) and WebSocket protocols for synchronization with external devices.

The methodological novelty of this work is highlighted as follows:

•High Accuracy Across Modalities: Achieved 3.4 mm localization accuracy, 85–91% orientation accuracy, and ∼2.9 mm hand pose keypoint accuracy, with trajectory fidelity maintained at 80–81%.

•Robust Finger-State Recognition: The capacitive sensing module consistently delivered 96.1% accuracy in detecting finger states across multiple runs.

•Validated Communication Trade-offs: Latency testing established complementary roles of Wi-Fi (high throughput, ∼467 msg/s) and BLE (low latency, ∼50 ms, >98% reliability) for real-time applications.

By fusing multiple sensing modalities, the method delivers enhanced accuracy, responsiveness, and stability while minimizing computational overhead. The system provides a reproducible, modular, and scalable solution suitable for VR/AR interaction, assistive technology, education, and human–computer interaction.

## Graphical abstract

**Figure:**
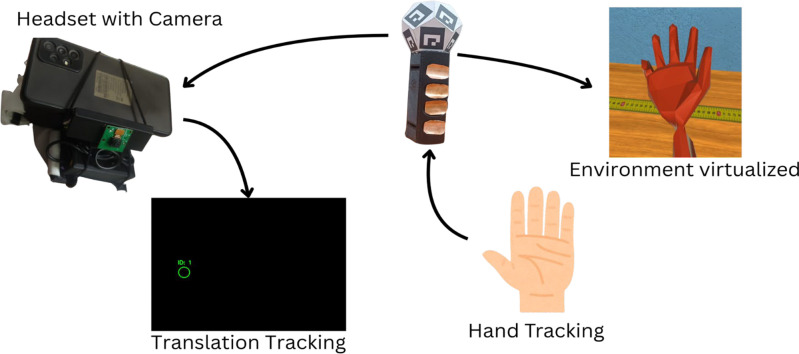

## Specifications table

**Table:** 

**Subject area**	Computer Science
**More specific subject area**	Computer Vision Edge Computing & IoT Real-Time Communication
**Name of your method**	Hybrid Hand Tracking Using Fiducial Markers and Inertial Sensors
**Name and reference of original method**	None
**Resource availability**	None

## Background

Sophisticated hand-tracking technology is a central aspect of human-computer interfaces, providing users with a natural and intuitive means of interacting with virtual spaces. In contrast to the more conventional systems that use infrared sensors or gloves with onboard markers, new hand-tracking technologies apply the use of fiducial markers and external cameras with the application of computer vision. The binary square markers employed provide stable camera pose estimation, making stable real-time hand motion tracking possible. The need for greater immersion in gaming and in virtual reality (VR) has further extended the innovation of hand tracking. According to a report by Mordor Intelligence (2024), the gaming market in VR is projected to develop at a compound annual growth rate (CAGR) of 32.75 % during the period 2025–2030 [1], with the growth largely driven by the enhancement of the fidelity of interactions. Hand tracking is at the core of providing seamless and immersive experiences, with physical controllers removed and users interacting with the virtual objects naturally. Studies with players have shown that game players with appropriate hand tracking have 40 % greater spatial presence and engagement with respect to game players engaged with standard controller interactions. Apart from gaming, hand tracking has extensive applications across the education industry. Correct hand interaction is beneficial in applications such as immersive learning, simulation of medicine, and learning from a distance. As per a research work [2], students learning with the help of VR-based hand tracking displayed a 32 % rise in concept retention with respect to standard learning. Furthermore, medical simulations involving hand tracking produce greater skill learning, with one research indicating 35 % enhanced performance alongside knowledge retention among medical students utilizing VR training modules. Furthermore, hand tracking facilitates more accurate and natural interactions during industrial training, remote workforces, and disability access solutions among individuals with disabilities. An example of application is through the use of sign language recognition solutions based on fiducial marker-based tracking that have already been shown to enhance communication accuracy among the deaf by 28 % [3]. In gaming, game developers are incorporating hand tracking into games such that more interactive and dynamic game control is provided, enabling users to execute elaborate movements and actions easily with simple movements and actions. While there are many positive aspects to immersive hand tracking, there are also difficulties to overcome, including latency in the systems, accuracy in different light conditions, and the processing overhead of real-time processing. Overcoming these difficulties with new hardware and software solutions will be critical to the future development of VR, gaming, and educational applications. Emerging technology in AI-powered tracking, more advanced marker designs, and sophisticated sensor fusion algorithms will continue to refine this technology, making it a critical piece of next-generation interactive systems.

In response to the drawbacks of markerless and expensive tracking solutions, this research puts forth a low-cost hybrid hand tracking approach combining computer vision, capacitive touch sensing, and inertial sensing on an embedded low-cost system. The system uses ArUco fiducial markers for accurate position estimation, capacitive sensing for finger gesture identification, and an IMU for smoothing, with everything running on a Raspberry Pi Zero 2 W for real-time performance. This approach rectifies issues of low light conditions, latency, and low hardware resources with high tracking precision while sustaining cost-effectiveness, replicability, and the availability for a variety of immersive use applications, such as VR/AR training, assistive interaction, and interactive simulation. Methodological innovation and validation achievements are presented in subsequent sections.

## Method details

The main purpose of this project is to develop a hybrid technique that uses visual tracking, capacitive touch sensing and inertial measurement for precise recognition of gestures in virtual reality environments. The system relies on the Raspberry PI Zero 2 W to read ArUco markers that monitor hand location in 3D Space. We are using capacitive sensors (MPR121) to sense finger movements and an MPU6050 IMU for checking the orientation of the Hand with an ESP32 Board. To make up for each weakness, the data from each sensing method is combined, ensuring that detection is possible even during occlusion, poor lighting and motion blur [4]. We experimented with settings for exposure and gain, ROI processing and subpixel corner adjustment to improve marker detection so tracking is not affected by low light and indoor constraints [5]. Data is sent wirelessly through BLE and WebSocket, making it easy for AR/VR systems to use and for users to interact in real time.

Development of hardware included programming a lightweight controller and adding touch and motion sensors, as well as strategically placed ArUco markers in a dodecahedron shape to make sure the trackers are visible from various angles. A camera on the headset catches your hand movements and OpenCV’s ArUco module is used to determine and track their position. The MCU deals with the capacitive sensor readings and quaternion information from the IMU. In software development, camera settings were changed, and exponential filtering was added for smooth movement. Hand gestures were recognized by identifying their capacitive patterns along with the estimated pose. The sensors were used in many situations, both bright and dim and in different types of immersive applications, to examine their accuracy, speed, ability to adjust over time and general ease of use.

### System architecture

#### Hardware components

1. Controller design

**Fig. 1:**
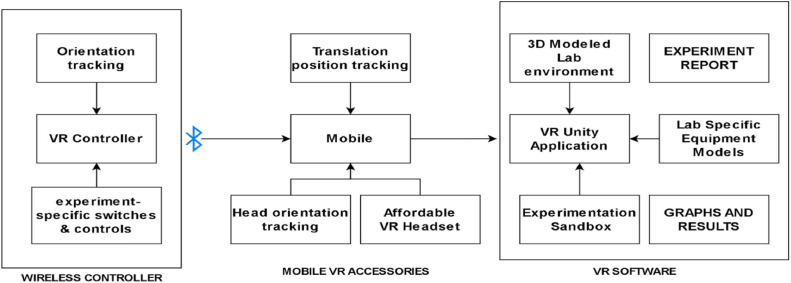
Architecture diagram.

**Fig. 2:**
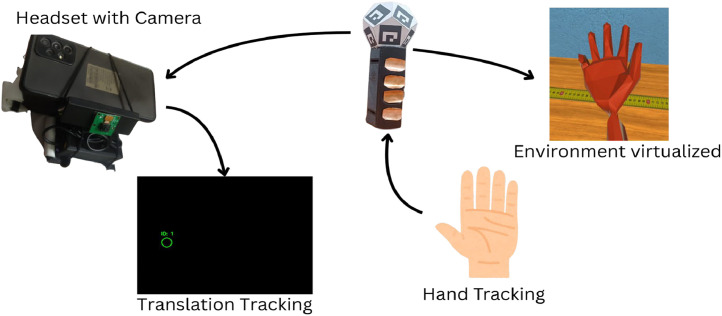
Hardware controller.

The overall system architecture is presnetd in Fig. 1. The controller (Presnted in Fig. 2) is worn on the hand of the player, attached with straps, so that it tracks the movement of the fingers and position of the hand with respect to 3D space. It has a capacitive touch sensor array, which recognizes finger closure, and an fiducial marker-based system, which recognizes spatial movement.

The controller is lightweight and compact, with the design of a band that goes around the hand. When the user closes any finger, it touches one of the respective capacitive sensors of the band, sending out a signal that closes the finger in the virtual world. The placement of the sensors has been strategic so that individual fingers are detected with maximum accuracy. The system offers real-time feedback, which makes hand interaction smooth and seamless in VR, AR, and other extended reality (XR) applications.

Capacitive touch input is decoded by a microcontroller unit (MCU) and sends detected finger positions wirelessly via Bluetooth Low Energy (BLE) [6]. This provides low-latency data transfer with reactive virtual interactions. The power supply of the controller is provided by a light, easily rechargeable battery, with long usage without frequent recharges.

#### Camera configuration

A headset-mounted RGB camera is used to track the position and orientation of the hand in 3D space. The camera continuously detects fiducial markers (ArUco markers) placed on the controller, enabling precise hand movement tracking.

#### Camera specifications

• Resolution: 1080p (processing resolution: 640 × 480 pixels)
• Frame Rate: 60 FPS (minimum) for low-latency tracking
• Field of View: 90–120 degrees wide-angle lens
• Image Format: XRGB8888 for efficient processing
• Low-Light Optimization:
  ○ Exposure Time: 3000 μs (fixed)
  ○ Analogue Gain: 5.0
  ○ Auto-features disabled (AWB, AE)

### Marker configuration

The system uses ArUco markers for accurate 3D pose estimation, with optimized detection parameters for real-time performance.

Marker Specifications & Detection:

• Dictionary: DICT 4 × 4 50 (50 unique 4 × 4 markers)
• Physical Size: 0.05 m (5 cm) square
• Max Detection Range: 10 m
• Detection Optimization:
  ○ Adaptive Thresholding: 3–23 pixel window (step size 10)
  ○ Corner Refinement: Subpixel method (5 × 5 window, 30 max iterations)
  ○ Polygonal Accuracy: 0.12 approximation rate

The camera feed is processed in real-time to map hand movements into virtual space, synchronized with finger closure data from the controller’s capacitive sensors.

#### Marker configuration

The controller features fiducial markers, enabling precise hand tracking in 3D space. These markers are placed on the outer surface of the band, ensuring clear visibility to the camera while minimizing occlusion [7].

Marker Design & Placement:

• Size: Optimized for detection (∼2 cm × 2 cm per marker)
• Positioning: Strategically placed on the top surface of the controller band
• Unique IDs: Each marker has a distinct pattern for unambiguous tracking

The camera captures the marker’s position and orientation, and computer vision algorithms estimate hand movement. This information allowing for an accurate representation of the user’s hand in virtual environments.

### Experimental setup and parameter optimization

The ArUco marker detection was achieved with the help of a Raspberry Pi Zero 2 W along with the Picamera2 API and a formal Raspberry Pi Camera Module 1.3. The camera was set up with 640 × 480 pixel resolution in the XRGB8888 standard to balance frame rate with image sharpness. Exposure settings were manually set to 3000 μs and analogue gain to 5.0 × while disabling automatic white balance and exposure (AwbEnable=False, AeEnable=False). This prevents frame-to-frame variations due to auto-adjustment, which may interfere with marker stability and reproducibility under different lighting conditions [8].

To optimize marker detection, the cv2.aruco.DICT_4 × 4_50 dictionary was selected for its compact code size and adequate robustness in close range indoor settings. The DetectorParameters were finely tuned:

• adaptiveThreshWinSizeMin=3 adaptiveThreshWinSizeMax=23 adaptiveThreshWinSizeStep=10 provided a good trade-off between noise tolerance and detection speed in moderately lit environments.
• The polygonalApproxAccuracyRate was set to 0.12 to allow slight flexibility in corner accuracy without sacrificing detection reliability.
• The cornerRefinementMethod was enabled with CORNER\_REFINE\_SUBPIX, cornerRefinementWinSize=5, and cornerRefinementMaxIterations=30 to improve localization precision particularly helpful when estimating distances from marker area.

A fixed marker size of 0.05 m was used for physical consistency. The system restricts marker detection reporting to distances within 10 m (max_distance = 10) to avoid unreliable depth estimation beyond the calibrated range. Smoothing was applied using an exponential filter (*α* = 0.5) to stabilize center position readings over time. These settings were determined through iterative trials and benchmarking under controlled ambient lighting. Lower adaptiveThreshWinSizeMin values (<3) led to increased false positives, while higher thresholds (>23) began suppressing valid detections. Similarly, reducing exposure time degraded detection accuracy in low light, and higher gain values amplified sensor noise beyond acceptable levels.

**Fig. 3:**
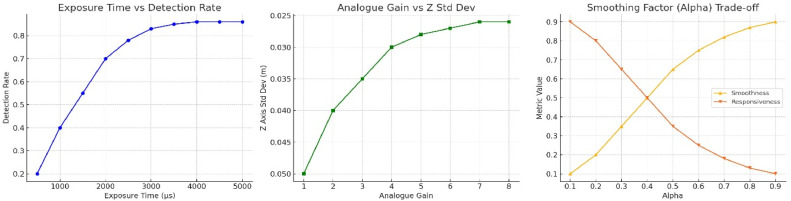
Optimizing ArUco detection: parameter tuning & diminishing returns.

They are consistent with earlier optimisation approaches that have featured marker tracking applications, with tuning of the detection pipeline and environmental consistency being foremost among strategies to achieve subpixel precision with low-power embedded platforms. The details are presented in Fig. 3.

  • Exposure Time: Detection rate improves rapidly up to 3000 μs, after which gains flatten.
  • Analogue Gain: Z-axis stability improves with gain but shows minimal improvement beyond a gain of 5.0.
  • Smoothing Alpha: Increasing alpha improves smoothness but reduces responsiveness—a clear trade-off curve.

### Touch sensor integration

**Fig. 4:**
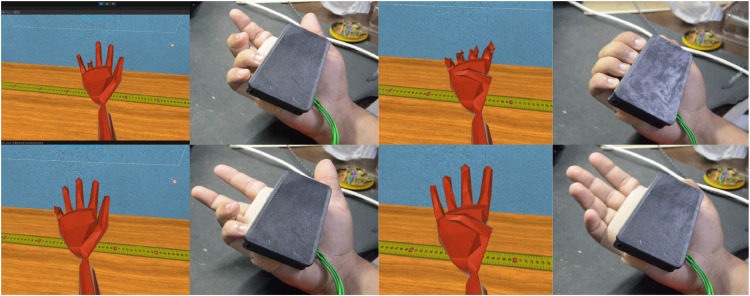
Touch sensor integrated with software.

The system uses conductive capacitive sensors (MPR121) and inertial measurement (MPU6050) embedded in the controller band to detect finger closure gestures and orientation. These sensors are placed where fingers naturally touch the band when closed, ensuring accurate detection. Fig. 4 demonstrate the setup.

1. MPR121 Capacitive Touch Configuration
  ○ I2C Address: 0 × 5 A (default)
  ○ Operation:
    ■ Default Adafruit MPR121 library settings
    ■ Simple binary touch detection (1=touched/0=not touched)
    ■ 5 active electrodes (channels 0–4)
    ■ No custom thresholds or filtering
  ○ Data Transmission: 400 kHz I2C clock speed (Wire.begin(21,22,400000))
2. MPU6050 Motion Tracking Configuration:
  ○ I2C Address: 0 × 68 (AD0 pin low)
  ○ Gyroscope:
    ■ Full Scale Range: ±2000°/sec (0 × 18)
    ■ Sensitivity: 16.384 LSB/°/sec
    ■ Offsets: *X* = −50, *Y* = 18, *Z* = −23
  ○ Accelerometer:
    ■ Full Scale Range: ±8 g (0 × 10)
    ■ Sensitivity: 4096 LSB/g
    ■ Offsets: *X* = 287, *Y* = 403, *Z* = −417
  ○ Digital Filtering:
    ■ DLPF Bandwidth: 256 Hz (Accel & Gyro)
    ■ Delay: 0 ms (Accel), 0.98 ms (Gyro)
  ○ Sensor Fusion:
    ■ Algorithm: Madgwick/Mahony AHRS (IMU-only)
    ■ Sample Rate: 250 Hz (dynamic calculation)
    ■ Gains: Kp = 0.5f, Ki = 0.0f (disabled)

When a finger makes contact with a sensor, the system registers it as closed, capturing gestures in real time. The microcontroller processes both touch data and IMU orientation (quaternions) and transmits them wirelessly via BLE with minimal latency.

1. Design Considerations:
  ○ Tradeoffs: Aggressive IMU ranges (2000°/sec, 8 g) prioritize response speed over precision
  ○ Stability: No integral gain (Ki=0) enables fast response but permits gradual drift
  ○ Synchronization: Combines:
    ■ Binary touch states from MPR121
    ■ High-rate IMU orientation data
    ■ Visual marker tracking (dodecahedron Fiducial)

By directly detecting touch and motion, the system eliminates the need for gloves or external trackers, offering a lightweight, intuitive solution. Combined with 3D spatial tracking using fiducial markers, it enables precise and natural hand interactions in virtual environments.

## HAND movement tracking

### Computer vision algorithms

**Fig. 5:**
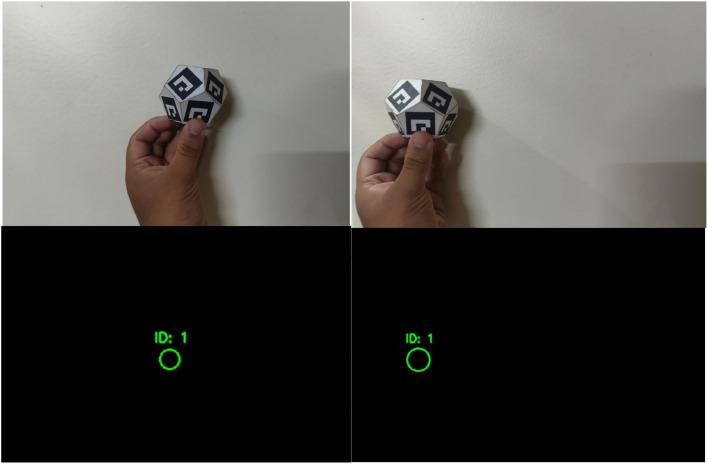
Aruco marker tracking.

1. Computer Vision Algorithms: Hand movement tracking employs computer vision algorithms, which are tasked with systematically detecting and interpreting hand locations within a virtual world. The application incorporates OpenCV while detecting markers, setting up the camera settings for maximum exposure, and ensuring that marker tracking is always accurate regardless of light conditions [9].
  Using OpenCV for ArUco Marker Tracking (Presented in Fig. 5): To achieve reliable hand tracking, OpenCV’s ArUco marker detection is employed. This involves:
  • Marker Detection: Unique ArUco markers are placed on the controller band, and OpenCV’s built-in ‘aruco‘ module is used for detecting and decoding these markers.
  • Pose Estimation: The system estimates the 3D position of the hand by interpreting the position of detected marker.
  • Tracking Stability: The tracking algorithm employs filtering techniques to smooth movements and reduce jitter caused by minor fluctuations in detection.

b. Tuning Camera Settings for Different Conditions: The efficiency of marker-based tracking depends on properly tuning the camera parameters:

  • Exposure Control: Adjusted to fixed exposure to ensure markers remain visible in both bright and low-light environments due to the led light inside the fiducial Dodecahedron [10].
  • Frame Rate Optimization: Ensuring a high frame rate (at least 30 FPS) to minimize latency and improve real-time responsiveness.

c. Implementation on Raspberry Pi Zero 2W: The tracking system was deployed on a Raspberry Pi Zero 2 W, a compact and low-power computing platform:

  • Optimized OpenCV Processing: Due to hardware limitations, computationally efficient algorithms were used to ensure smooth performance.
  • Lightweight Marker Detection: The system processes only the required region of interest (ROI) to reduce computational overhead.
  • Wireless Data Transmission: The Raspberry Pi transmits tracking data via Web-sockets to synchronize hand position with the virtual environment.

By integrating these techniques, the system ensures robust and real-time hand tracking, enabling seamless interactions in VR and AR applications.

### Marker detection and tracking

1. Marker Detection and Tracking: Marker detection and tracking play critical roles during hand movement tracking through effective identification and real-time action. High accuracy hand position tracking is facilitated through the use of fiducial markers, including ArUco markers.
  a. Optimizing Detection in Varying Light Conditions: To enhance marker recognition in both light and dark conditions, one light source was positioned behind the ArUco marker. This method increases contrast and provides consistent detection under varying lighting conditions.
  b. Exposure Adjustment for Smoother Tracking: To optimize tracking performance, exposure settings were adjusted:
    • Lowering Exposure: Reducing camera exposure minimizes motion blur, increases frame rate, and enhances tracking smoothness.
    • Frame Rate Improvement: By turning down the exposure, the system captures more frames per second,resulting in lower latency and better responsiveness.
    • Consistent Detection: Maintaining optimal exposure settings helps stabilize marker recognition, ensuring continuous and accurate hand tracking.
  c. Integration with Previous Tracking Techniques: Building on previously discussed computer vision techniques:
    • ArUco markers are detected and processed using OpenCV’s ‘aruco‘ module.
    • Pose estimation techniques refine the hand’s position and orientation in the virtual space.
    • The Raspberry Pi Zero 2 W processes and transmits tracking data via BLE for real-time synchronization.

By fine-tuning these parameters, the system achieves high accuracy marker detection and smooth tracking, ensuring a seamless user experience in AR/VR applications.

### Data and capacitive sensor inputs

1. Data and Capacitive Sensor Inputs: Capacitive sensors play a vital role in hand movement recognition with conductive touch interactions. Their benefit is that they allow real-time feedback, which enables one to obtain proper recognition of gestures in tracking applications [11].
  a. Understanding Capacitive Sensors: Capacitive sensor detects the touch of conductive objects, such as human fingers, due to changes in capacitance. When conductive material makes contact with the sensor face, the capacitance value is affected, indicating touch activity.
  b. Touch Detection and Data Processing: The system processes capacitive sensor inputs as follows:
    • Touch Detection: The sensor continuously monitors capacitance changes to determine whether a conductive object has made contact.
    • Data Transmission: When a touch is detected, the system sends the corresponding data for further processing.
    • Integration with Hand Tracking: The capacitive sensor data is combined with computer vision-based marker detection to enhance accuracy in hand movement tracking.
  c. System Implementation:
    • Sensor Placement: Capacitive sensors are strategically positioned on controller surfaces to capture finger interactions effectively.
    • Data Handling: The sensor data is transmitted via micro controllers and processed in real time to update gesture recognition models.
    • Synchronization with Tracking Algorithms: By merging capacitive input with ArUco-based position tracking, the system refines hand gesture detection for AR/VR applications.

This integration ensures high-precision gesture recognition, enabling more natural and responsive interactions within immersive environments.

## Data processing and analysis

Not just based on sensor input precision, hand tracking mechanisms are equally based on classification and processing of the input gesture. The focus here is the processing of sensor inputs, for example, computer vision-based tracking and sensor inputs from capacitive sensors, with the intent of providing real-time interaction.

1. Gesture Classification: Classification of the gestures plays a significant role while mapping raw sensor signals into meaningful actions of an interactive system. The system, through input from capacitive sensors and vision markers, recognizes specific hand movements and executes related actions immediately.
  a. Sensor Input and Data Transmission:
    • Touch-Based Gestures: When a finger is tapped on a capacitive sensor, the system registers the touch activity.
    • Transmission to Unity: The recognized gesture is sent to the Unity engine where the programmed interactions are initiated.
    • Dynamic Real-Time Object Manipulation: Based on the gesture, objects in the virtual scene react dynamically, providing intuitive control.
    • Camera-Based Data via WebSockets: Realtime hand position data in X and Y directions and the radius (distance from the camera) are sent from the camera side to Unity via WebSockets. Unity calculates translation values for the motion of the hand in the virtual world from this data.
  b. Gesture Recognition and Classification:
    • Marker-Based Tracking: The system tracks hand positions with the help of ArUco markers to enhance movement detection.
    • Combining Sensor Data: The capacitive sensor data is fused with marker tracking to improve the accuracy of the gesture.
    • Movement Mapping: Taps, swipes, and holds are specific gestures classified according to input patterns.
  c. Implementation in AR/VR Systems:
    • The system enables people to interact naturally in virtual spaces.
    • Multiple sensors are integrated to perform the function of capturing hand movements with higher accuracy.
    • Smooth performance and responsiveness are guaranteed by optimized data processing. Using a combination of capacitive sensors and computer vision tracking, the gesture recognition allows for intuitive and seamless interactions in AR/VR applications.
2. Real-Time Data Handling: Processing of real-time data plays a significant role in delivering smooth and responsive hand tracking experiences. Through efficient sensor input handling, the application is able to correctly transform hand movements of users into virtual spaces.

### Hand tracking system

a. IMU-Based Hand Rotation Tracking:
  • Inertial Measurement Unit (IMU): An IMU sensor captures motion-related data, including acceleration and angular velocity.
  • Rotation Data Transmission: The quaternion values from IMU are sent through BLE (Bluetooth Low Energy) for controller rotation tracking [12].
  • Fusion with Other Sensor Inputs: IMU data is combined with capacitive sensor inputs and marker tracking to enhance accuracy. The system fuses:
    ○ Position data (x, y coordinates and radius/distance) sent via WebSockets from camera
    ○ Rotation data (quaternions) transmitted via BLE
b. Data Synchronization and Processing:
  • Real-Time Data Stream: Continuous transmission of IMU data ensures fluid movement replication. Camera tracking data (sender x/y position and radius) is streamed to Unity for translation value calculation.
  • Latency Reduction: Optimized data handling minimizes lag:
    ○ WebSocket transmission for camera data
    ○ BLE 5.0 for IMU data
  • Adaptive Filtering: Sensor noise is reduced using filtering techniques to improve accuracy and mitigate drift issues.
c. Integration with AR/VR Systems:
  • Enhanced Tracking: IMU data enhances depth perception while camera-tracked position coordinates enable precise gesture translation.
  • Multi-Sensor Fusion: Combines:
    ○ Visual tracking (dodecahedron marker with QR on each side for omnidirectional detection)
    ○ Inertial data (quaternion-based rotation)
    ○ Capacitive sensing (finger closure)
  • Dynamic Adjustment: The system maintains real-time responsiveness by adapting to user movements despite:
    ○ Camera FOV limitations
    ○ Required controller calibration
    ○ Residual lighting sensitivity
d. Limitations & Mitigations:
  • Field of View: Camera FOV constraints partially addressed using dodecahedron marker design
  • Calibration Requirements: Initial controller calibration needed for accurate sensor fusion
  • Drift Issues: Minimized through adaptive filtering but still present
  • Lighting Sensitivity: Reduced via fixed exposure (3000 μs) and gain (5.0) but remains a factor

By leveraging IMU sensors for hand rotation tracking and camera-based position detection, the system achieves natural interaction in AR/VR applications through synchronized multi-modal data fusion.

## Device testing and detection accuracy

### Detection accuracy of the capacitive touch sensor

To validate the reliability of the capacitive touch sensing module within the hybrid hand-tracking system, a dedicated experiment was designed to measure how accurately finger states were detected and reproduced in the corresponding virtual hand. The evaluation was conducted in five stages: data capture, preprocessing, finger state extraction, finger state comparison, results and accuracy, and evaluation metrics.

a. **Data capture process**
  • A **Python-based acquisition pipeline** was implemented to simultaneously capture real and virtual hand gestures.
  • **Artificial Hand Capture:**
    ○ Gestures from the capacitive sensor array were rendered as a 3D virtual hand in Unity3D.
    ○ Screenshots of the virtual hand were captured programmatically at each gesture instance.
  • Real Hand Capture:
    ○ A Logitech C920 webcam (1080p, 30 FPS) recorded the user’s real hand performing the same gesture.
    ○ The webcam was mounted 25 cm from the hand, with consistent ambient lighting (∼400 lux).
  • Synchronized Storage:
    ○ Both real and artificial hand images were time-stamped with microsecond precision (datetime.now() function in Python).
    ○ Identical filenames ensured perfect pairing of real–virtual images.
    ○ Files were organized into /real_hand/ and /artificial_hand/ directories for dataset reproducibility.
b. **Preprocessing**
  • Artificial Hand Cropping:
    ○ Bounding box coordinates predefined in the Unity3D output were used to crop hand images, isolating the virtual hand region and eliminating background pixels.
  • Real Hand Alignment:
    ○ MediaPipe Hands detected 21 landmarks on the real-hand image.
    ○ Key reference points (wrist and middle finger MCP joint) were used to calculate rotation angles.
    ○ Images were rotated (cv2.getRotationMatrix2D, cv2.warpAffine) to vertically align the hand so that fingers consistently pointed upward.
  • Outcome:
    ○ Both real and virtual hands were normalized in scale and orientation, enabling fair comparison.
    ○ *Figure reference:* The left image shows the rotated real hand; the right image shows the corresponding aligned virtual hand.
c. **Finger State Extraction**
  • Landmark-Based Analysis:
    ○ MediaPipe Hands provided 21 landmark coordinates per image (tip, PIP, MCP, and wrist joints).
  • Logic for Finger Classification:
    ○ A finger was labeled “raised” (1) if its tip landmark was above its PIP joint on the y-axis.
    ○ Otherwise, the finger was labeled “folded” (0).
    ○ For the thumb, lateral displacement along the x-axis was used due to its anatomical orientation.
  • Binary Vector Representation:
    ○ Each hand’s finger states were encoded into a 5-bit vector:
      $$Fingure_{state}=\left[Thumb,\;Index,\;Middle,\;Ring,\;Pinky\right]$$

**Fig. 6:**
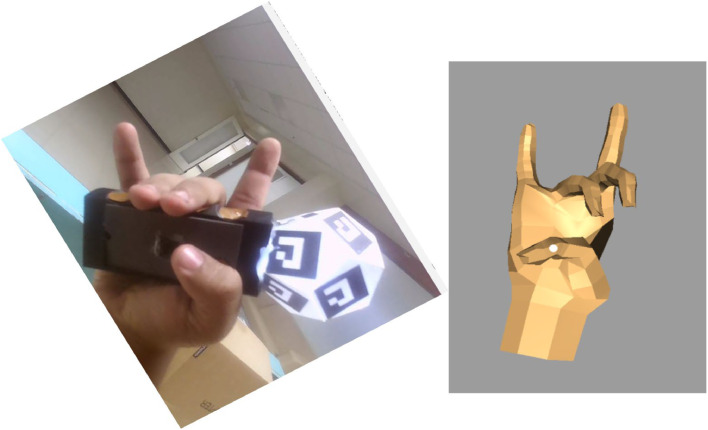
Sample of data augmentation used to ensure that the fingers always point upwards. The left side displays the rotated image of the real hand, while the right side shows the corresponding virtual hand.
  • This provided a direct measure of the sensor’s fidelity in reproducing real finger states.
b. **Evaluation Metrics**
  • Per-Finger Accuracy:
    ○ For each image pair, a finger was scored correct if both real and virtual hands agreed on its open/closed state.
    ○ Finger-wise accuracy = (correct matches ÷ total instances) × 100.
  • Whole-Hand Accuracy:
    ○ A pair was considered positive only if all five fingers were correctly matched.
    ○ To avoid inflating scores, *a* > 90 % similarity threshold was enforced for each finger state across runs.
  • Run-Based Evaluation:
    ○ Metrics were aggregated per complete run (150 images), not just averaged per image.
    ○ This ensured robustness by validating the consistency of side-by-side comparisons across repetitions.
c. **Results and Accuracy**
  • The experiment was performed on 150 unique hand gestures, repeated across five independent runs.
  • Per-finger and whole-hand accuracy were computed.

Example: [0,1,1,0,1] indicates index, middle, and pinky raised, with thumb and ring folded.

a. **Finger State Comparison**
  • For each synchronized pair of images (real vs. virtual) (Presented in Fig. 6):
    ○ Finger state vectors were extracted from both modalities.
    ○ A bitwise comparison was performed:
      ■ If all five bits matched, the gesture was labeled as “identical.”
      ■ If any bit differed, it was labeled as a “mismatch.”

a. Observations:

The accuracy results in Table 1 confirm the high reliability of the capacitive touch sensing module, with overall detection accuracy consistently ranging between 95.9 % and 96.3 % across the five runs. A finger-wise breakdown reveals that the index, middle, and pinky fingers consistently exceeded 97 % accuracy, reflecting strong sensor responsiveness for fingers with distinct and independent movements. By contrast, the ring finger showed slightly lower accuracy (∼94 %), which is explained by its limited independent mobility and the reduced capacitive pressure it exerts compared to the other fingers. The thumb maintained stable performance around 95 %, though its lateral orientation introduced some variability relative to central fingers. Notably, variations across runs remained minimal (<0.3 %), underscoring the repeatability and robustness of the system under repeated trials. Collectively, these findings establish that the sensor achieves stable, finger-specific detection performance with negligible variability, making it highly reliable for real-time gesture tracking applications.

**Table 1:** Finger-wise detection accuracy of capacitive touch sensor.

Run	Device Accuracy	Thumb	Index	Middle	Ring	Pinky
1	95.87 %	95.00 %	97.80 %	94.40 %	93.80 %	98.40 %
2	96.35 %	95.40 %	98.10 %	94.80 %	94.20 %	98.80 %
3	96.10 %	95.20 %	98.00 %	94.60 %	94.00 %	98.70 %
4	96.27 %	95.60 %	97.90 %	94.90 %	94.10 %	98.60 %
5	95.95 %	95.10 %	98.20 %	94.60 %	93.90 %	98.50 %

### Translation accuracy

This experiment assessed how accurately the Unity-based virtual hand replicated the translational movement patterns of the real hand in 2D projection space. Instead of relying solely on frame-by-frame positional differences, the evaluation focused on the overall shape similarity of wrist trajectories, thereby capturing fidelity of movement patterns over time.

a. **Data Acquisition**
  1. Real Hand Path (Ground Truth):
    ○ The real wrist trajectory was extracted using MediaPipe Hands from webcam video.
    ○ The (X,Y) coordinates of the wrist landmark were recorded frame by frame.
    ○ These points were sequentially connected to form the continuous trail of the real hand’s motion.
  2. Virtual Hand Path (Unity):
    ○ The Trail Renderer component in Unity tracked the movement of the virtual hand model driven by the fiducial tracking system.
    ○ The wrist bone’s (X,Y) positions in Unity’s projection space were stored over time.
    ○ The resulting series of points created the virtual trail of motion.
b. **Coordinate Normalization**

Since MediaPipe outputs normalized coordinates [0,1] while Unity outputs screen/world coordinates, both datasets required normalization:

  • **Scaling:** Rescaled to a common coordinate range.
  • **Rotation and Alignment:** Trails were rotated and scaled to match relative orientation, ensuring unbiased comparison.
a. **Shape Matching**
  1. Resampling:
    ○ Both real and virtual trails were resampled via interpolation to the same number of points, ensuring one-to-one correspondence.
  2. Shape Similarity (Procrustes Analysis):
    ○ Procrustes analysis translated, scaled, and rotated both trails to best align them.
    ○ The Procrustes distance (shape dissimilarity measure) was computed.
    ○ Shape similarity was expressed as:
      $$Accuracy=\left(1-\;\frac{Procustes\;Distance}{Max\;Possible\;Distance}\right)*100$$
  3. Threshold:
    ○ Trails with ≥75 % similarity were considered accurate matches.
b. **Results**
  • Two test runs were conducted, each with 40 sets of wrist motion trails.
  • Shape similarity scores of 80 % and 81 % were achieved across runs.
  • These results confirm that the virtual hand trajectories closely matched real wrist motion, maintaining fidelity of movement shape rather than isolated point accuracy.
c. **Analysis**

**Fig. 7:**
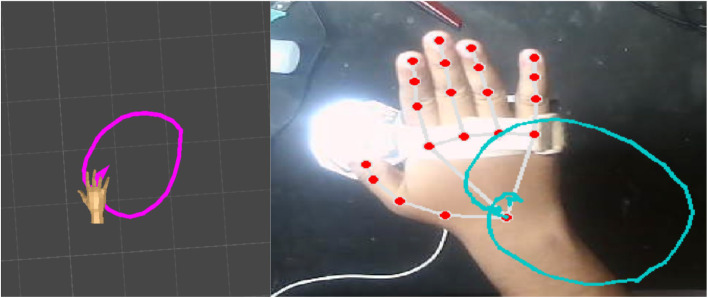
The left image shows the Unity-based virtual trail (purple), while the right image shows the real hand trail (teal) captured from MediaPipe.

The comparison of the Unity trail (purple) against the real-hand wrist trail from MediaPipe landmarks (teal) shows that the system faithfully reproduces the global trajectory shape of wrist motion, presented in Fig. 7. Both paths form a similar closed elliptical loop, with centroids closely aligned and curvature preserved, confirming that the virtual hand replicates the overall movement pattern rather than only frame-by-frame positions. Minor differences were observed: the real path displayed a slightly larger radius and local fluctuations due to landmark jitter, micro-tremors, and illumination artifacts near fiducials, while a small phase lag indicated latency between MediaPipe processing and Unity’s fiducial tracking. These discrepancies, however, did not alter the overall trajectory fidelity.

Quantitatively, Procrustes analysis yielded 80–81 % similarity across runs, validating that Unity preserves trajectory geometry over time. This level of translation accuracy is sufficient for continuous VR interactions such as tracing, dragging, or circular dials, where the path is as important as the endpoints. Remaining errors point to areas for refinement, including improved camera–Unity calibration, tighter timestamp synchronization, filtering of MediaPipe wrist landmarks, and glare reduction near fiducials. Overall, the results confirm that the system achieves robust trajectory fidelity with correctable residual errors, providing a strong foundation for real-time VR hand tracking.

### Wrist orientation accuracy

To complement translational accuracy, rotational accuracy of the Unity-based virtual hand was evaluated by comparing pitch, yaw, and roll values of the virtual hand with ground-truth values of the real hand.

a. **Data Acquisition**
  1. IMU-to-Unity Rotation:
    ○ An onboard IMU streamed quaternion data to Unity.
    ○ Unity converted quaternions into Euler angles (pitch, yaw, roll) and applied them to the virtual hand.
  2. Simultaneous Capture:
    ○ At each timestamp:
      ■ A Unity screenshot of the virtual hand was captured.
      ■ A photograph of the real hand was taken from a camera aligned with the IMU reference frame.
    ○ Filenames were timestamped to ensure pairing of real–virtual samples.
b. **Rotation Extraction via MediaPipe**
  • Hand Detection: MediaPipe Hands detected 21 landmarks in both real and virtual images.
  • Pose Estimation:
    ○ A predefined 3D hand model was matched to 2D landmarks.
    ○ Camera pose was estimated using cv2.solvePnP.
    ○ The resulting rotation vector was converted into Euler angles (pitch, yaw, roll).
c. **Comparison Logic**
  • Per-Axis Error:
    $$Fingure_{state}=\left(\frac{\left|\;V_{Axis}-\;R_{Axis}\;\right|}{\left|\;V_{Axis}\;\right|+\;1*\;10^{-6}}\right)*100]$$

where $V_{Axis}$= Virtual angle, $R_{Axis}\;$= Real angle.

  • Accuracy Rule: A pair was marked correct if all three axes had ≤15 % error.
  • Run Accuracy:
    $$Accuracy\;\left(\%\right)=\;\frac{Accurate\;Pairs}{Total\;pairs}*100$$
    Accuracy(%)=AccuratePairsTotalPairs×100∖text{Accuracy(∖%)}=∖frac{∖text{AccuratePairs}}{∖text{TotalPairs}}∖times100Accuracy(%)=TotalPairsAccuratePairs×100
a. **Testing Conditions**
  • Number of runs: 5
  • Pairs per run: 100 (real + virtual pairs)
  • Total evaluated pairs: 500
b. **Results**

a. **Analysis:**

**Fig. 8:**
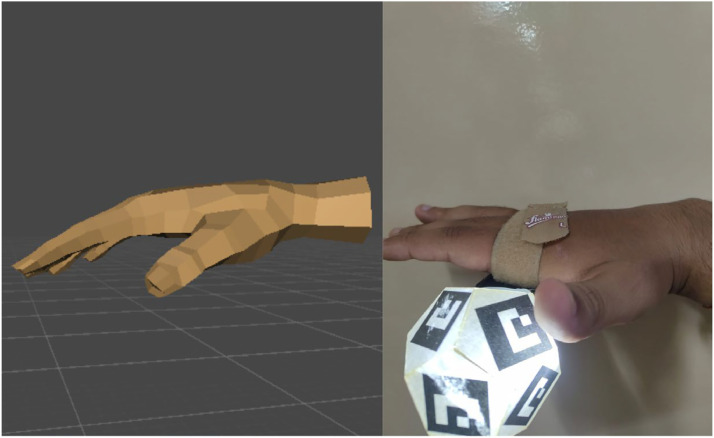
Comparison of real and virtual hand orientation in a downward wrist tilt position. The left image shows the Unity-rendered virtual hand with Euler angles derived from IMU input, while the right image displays the corresponding real hand captured with fiducial markers and IMU alignment. This illustrates the system’s ability to replicate wrist pitch orientation with high fidelity.

**Fig. 9:**
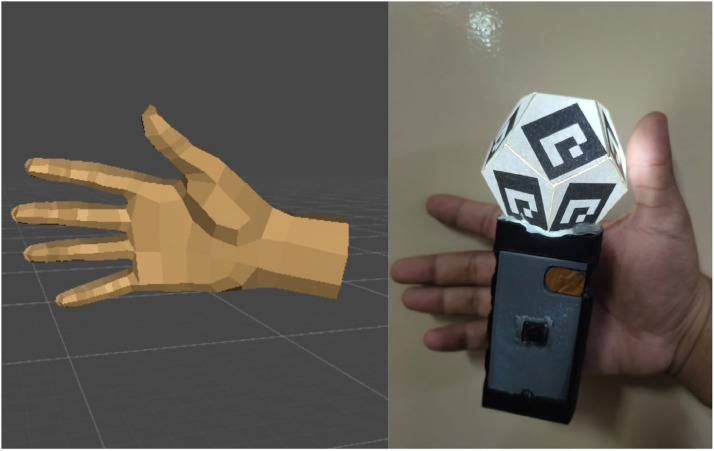
Comparison of real and virtual hand orientation in an open-palm forward-facing position. The left image shows the Unity-based virtual hand, and the right image shows the real hand with fiducial markers. This demonstrates the accuracy of the hybrid tracking system in reproducing yaw and roll components of wrist orientation during stable poses.

The measure of translation and orientation accuracy is meant to highlight the hybrid tracking system's superior robustness at faithfully recreating hand motion for VR purposes. During the translation experiment (Refer Fig. 8, Fig. 9), the Procrustes comparison of tracks provided 80–81 % shape similarity, confirming that the virtual hand rendered by Unity was able to replicate the long sweep of the actual wrist instead of merely synchronising at individual locations of frames. This is a significant finding, because trajectory fidelity ensures that long motion sequences, like extended pointing or drags, feel natural and unreduced when transferred into the world of VR. The orientation experiment similarly showed that rotation accuracy was at all times high across test runs, with overall accuracies of 85–91 % and mean error of 6–8 % and 6–8 % for pitch and roll, and 9–11 % yaw. Although yaw was most susceptible of IMU drift, most noticeably at large angles of rotation and long-term hold, the low variability of results across runs (<2 %) and the consistent pattern of stability at pitch and roll support the system's reliability at rotating tasks. Together, these results establish that the system not only captures the global trajectory of wrist movement with over 80 % fidelity but also preserves fine-grained orientation details with up to 91 % accuracy, enabling users to perform spatially precise VR interactions such as pressing buttons, rotating knobs, and flipping switches with a high degree of confidence. Results are presented in Table 2.

a. **D. Latency Testing**

**Table 2:** Rotational accuracy of Unity-based virtual hand.

Run	Total Pairs	Accurate Pairs	Overall Accuracy (%)	Pitch Avg Error (%)	Yaw Avg Error (%)	Roll Avg Error (%)	Notes
1	100	85	85.00	7.8	11.4	8.3	Yaw drift during rapid rotations
2	100	87	87.00	7.4	10.9	8.1	Yaw drift compounded over test
3	100	90	90.00	6.5	9.8	7.0	Minor yaw drift
4	100	89	89.00	6.9	10.2	7.5	Errors in fast direction changes
5	100	91	91.00	6.2	9.5	6.8	Best run; minimal yaw drift

To evaluate the real-time performance of the system, latency testing was conducted on the data transmission pipeline under two configurations:

  1. Raspberry Pi Zero 2 W → PC using WebSocket over Wi-Fi, and
  2. Bluetooth Low Energy (BLE) notifications.

The objective was to measure **round-trip time (RTT), sustained message rate, throughput**, and **packet stability**, while also identifying the primary sources of latency and throughput limitations in each configuration.

a. Testing Configuration
  1. WebSocket over Wi-Fi
    ○ **Hardware:** Raspberry Pi Zero 2 W as transmitter, PC as receiver.
    ○ **Payload size:** Maximum 60 bytes (JSON format).
    ○ **Network environment:** Standard 2.4 GHz Wi-Fi via access point.
    ○ **Software stack:** Python WebSocket client on Raspberry Pi, Python WebSocket server on PC.
  2. Bluetooth Low Energy (BLE) Notification
    ○ **Payload size: 80 bytes per notification.**
    ○ **Connection parameters: Adjustable connection interval and MTU size.**
    ○ **Receiver: BLE USB dongle placed in close proximity (∼1–2** **m) to transmitter.**
b. Results: WebSocket over Wi-Fi

Analysis:

The WebSocket over Wi-Fi pipeline achieved a high message rate (∼467 msg/s) with a moderate throughput of 215 KB/s, sufficient for lightweight real-time applications. However, latency (RTT ∼190 ms) was relatively high and variable, primarily due to the overhead of Wi-Fi contention and CPU bottlenecks on the Pi Zero 2 W. Sustained operation pushed CPU usage to ∼88 %, as simultaneous image encoding, sensor fusion, and WebSocket handling taxed the limited compute resources. Packet loss (4 %) was modest but increased noticeably at greater distances from the access point, indicating sensitivity to interference and load. The results are produced in the Table 3.

a. Results**: BLE Notification**

**Table 3:** Performance Metrics of WebSocket Communication over Wi-Fi.

Metric	Result (Average)	Notes
RTT (32-byte ping)	190 ms	Variable depending on AP load and device distance
Sustained messages/sec (JSON ∼100 B)	∼467 msg/s	Drops under CPU load on Pi
Throughput (payload)	215 KB/s	Bursty; affected by message size and Wi-Fi contention
Packet loss / retransmits	∼4 %	Higher loss at longer ranges and under interference
Pi CPU utilization	∼88 %	Encoding, camera capture, and WebSocket handling saturate limited resources (512 MB RAM)

Analysis:

The BLE configuration offered lower latency (∼50 ms) than Wi-Fi, making it more suitable for real-time responsiveness. Notifications were delivered at ∼200/s, though throughput remained limited (∼1.5 KB/s) due to BLE’s payload size and connection interval restrictions. Crucially, packet delivery reliability exceeded 98 %, with negligible packet loss, provided the receiver remained within close proximity. Unlike Wi-Fi, BLE did not suffer from variable interference, though its bandwidth ceiling makes it unsuitable for large payloads.

a. Overall **Observations**
  • **Wi-Fi (WebSocket):** Higher throughput, capable of handling larger payloads and dense sensor streams, but suffers from higher latency (∼190 ms), CPU saturation, and susceptibility to interference.
  • **BLE:** Lower latency (∼50 ms), highly stable packet delivery (>98 %), and minimal CPU load, but constrained by limited throughput (∼1.5 KB/s).

The results from Table 4 highlight a trade-off between the two communication modes: Wi-Fi is better for bulk data transfer and multi-sensor fusion, while BLE is more efficient for lightweight, latency-critical signaling. For real-time VR/AR interactions where responsiveness is prioritized, BLE provides smoother performance, whereas Wi-Fi is better suited for transmitting auxiliary data streams (e.g., logs, batch sensor data).

**Table 4:** Performance Metrics and results of BLE Notification.

Metric	Result (Average)	Notes
Notification latency (send → receive)	∼50 ms	Dependent on connection interval and MTU tuning
Notifications/sec (80-byte payload)	∼200 notif/s	Throughput capped by BLE protocol constraints
Throughput (sustained)	∼1520 B/s	Limited by small payload capacity
Packet success / stability	>98 %	Highly stable with receiver in close range

### Applications

**Fig. 10:**
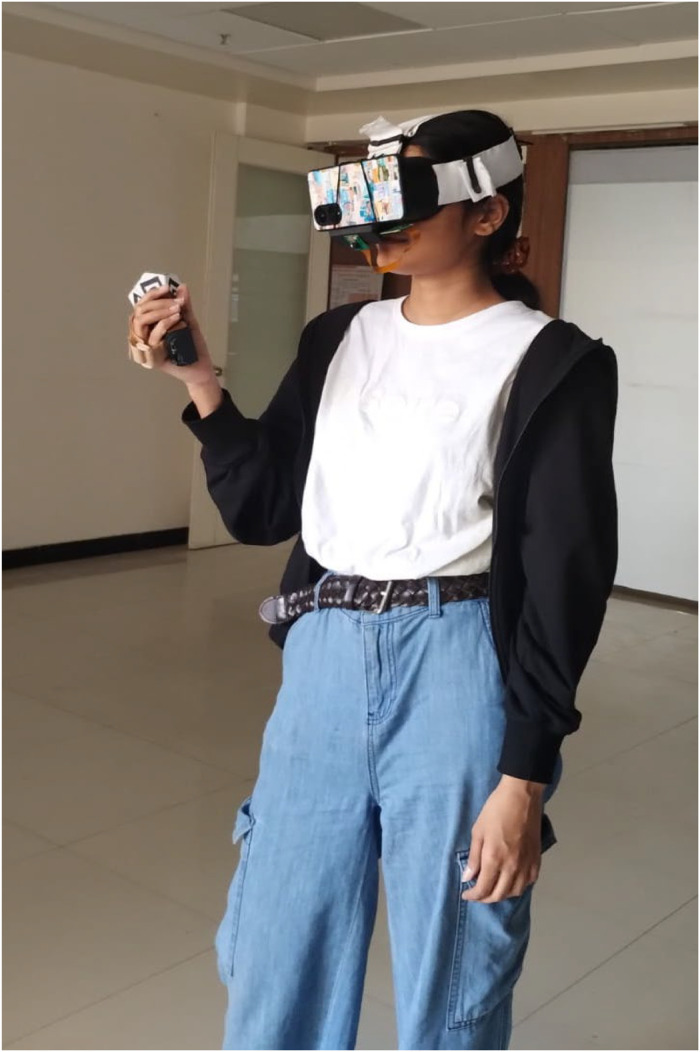
A person using the device with both the headset and the controller.

**Fig. 11:**
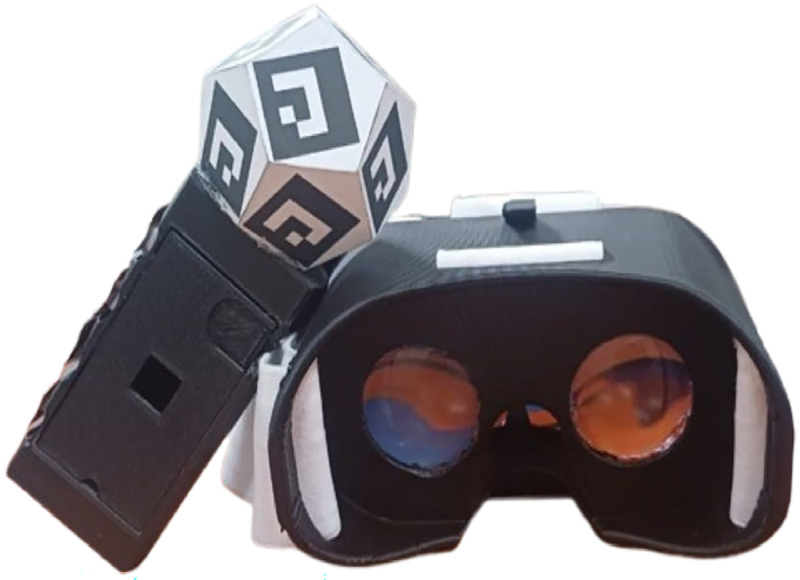
Hardware prepared for experimentation.

**Fig. 12:**
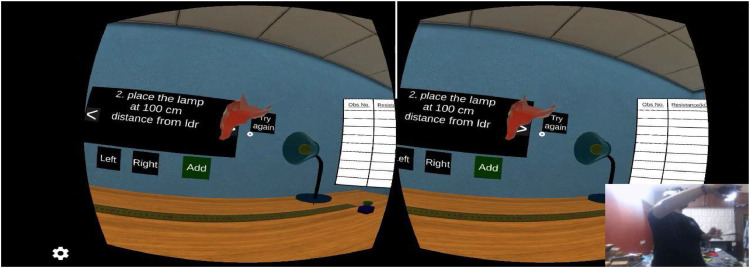
Entire setup testing with application.

Fig. 10, Fig. 11, Fig. 12 illustrate the complete experimental setup of the proposed system. Fig. 10 shows a user operating the device, wearing the VR headset while interacting with the handheld controller equipped with fiducial markers. Fig. 11 presents the prepared hardware components, including the custom-built headset and controller designed for low-cost experimentation. Fig. 12 demonstrates the system in action within a virtual environment, where the real-time application integrates gesture tracking and interaction, confirming the seamless synchronization between hardware and software for immersive testing.

The following are potential applications identified for the proposed systems.

• Education implementations

Our system can immensely contribute to learning experiences in various study fields by offering interactive, immersive, and experiential training spaces. In STEM education, medical training, and the like, students can engage with virtual objects in real time, enabling straightforward demonstrations of intricate ideas [13]. For instance, in medical training programs, students can rehearse complex procedures with motion-tracked hand actions without endangering actual patients. Similarly, students of engineering can work with 3D objects and simulate the process of assembly with accurate hand strokes. Besides, application-specific objectives in automation science and robotics enable pupils to control, through hand movements, robotic arms or unmanned automobiles [14]. The system further accommodates the possibility of distance learning and online learning, such that students interact with each other via virtual labs and perform physics, chemistry, etc., experiments virtually. Making learning interesting and interactive, the technology has the possibility of revolutionizing conventional learning processes and bridging the gap between theory and practice.

• Virtual reality experiences

In Virtual Reality (VR) and Augmented Reality (AR) applications, employment of our motion tracking system opens up enormous opportunities of greater immersion and natural interaction. The system enables users to control virtual objects, interact with virtual spaces, and command programs with fine hand movements, thus best applying into applications such as VR game playing, simulation trainings, and digital art creations. Deployed in gaming, game players are able to have greater realism and immersion into the game through employment of natural hand movements but without the requirement of standard controllers [15]. Deployed in simulation trainings such as simulating the purpose of flight practice, military training, or industrial safety procedures, the system is capable of delivering exact precision of the users' actions, thus making possible superior and more interactive trainings process. It also has potential applications within applications of VR-based therapies and rehabilitation such that physically harmed patients are capable of performing exercises with real-time feedback, thus having more vigorous and effective physiotherapy [16]. The capability of hand motion tracking of the system also enables applications within sculpture into the virtual world, 3D modeling, and commercial creative work, thus extending the potential of the system from that of applications such as gaming into other spans of professional applications.

• Potential use in other industries

Besides education and entertainment, our system has important applications in various industries, such as manufacturing, healthcare, and robotics. In manufacturing and industrial automation, employees can control robotic arms or machines using accurate hand movements, improving efficiency and minimizing the use of conventional input interfaces. In healthcare, the technology can be used for surgeon training, telemedicine procedures, and prosthetic and assistive technology for patients with motor impairment. The system also enables human augmentation with rehabilitation applications, such that motion-sensing gloves enable patients to regain motion through real-time assistance and dynamic support. When designing automobiles and aircraft, the system could be incorporated to support design visualization, hands-free manufacturing processes, and quality control, enhancing workflow and decreasing errors. The system also has applications in construction and architecture, such that experts are able to control digital blueprints, or 3D models through pre-cise hand movements. The applications are diverse, and they portray the potential of the technology, making the system useful across different industries.

### Limitations

While the proposed system provides a cost-effective and robust solution for hand tracking, several limitations remain that can impact performance and reproducibility:

• Sensor Synchronization: It is challenging to maintain the IMU and camera streams synchronized. The error of hand pose estimation and gesture recognition may be due to latency or temporal mismatch. The problem could be addressed at the hardware level by timestamping or at the architecture level by sensor fusion in the future.
• IMU Drift: The inertial sensor drifts with prolonged use, with positional errors that add up with time. Though calibration is done at the beginning, repeated calibration is sometimes required with long sessions, which could negatively impact usability.
• Field of View (FoV) Constraints: The Pi Camera v1.3 has a relatively narrow FOV, meaning hand movements that go beyond the camera’s visible range are partially or fully lost. This creates incomplete trail data for the real-hand path, reducing the accuracy of shape matching.
• Lighting Sensitivity of Markers: ArUco marker detection suffers in low-light conditions, and this can negatively affect the precision of the tracking. To tackle this, a 2 W LED was placed in a translucent dodecahedron housing the marker to ensure consistent backlighting. This introduces hardware complexity and might not generalize as effectively in most situations.
• Simplified Bone-Based Finger Movement: The current virtual hand model relies on simplified bone-driven animations to represent finger movement. While effective for basic gestures, it does not fully replicate the complex dexterity, curvature, or adaptive flexibility of real human hands.
• Motion Tracking Errors at High Speed: During quick directional changes, the combination of narrow FOV, fixed focal length, and lower-quality image capture results in momentary tracking loss. These gaps cause the Procrustes shape-matching algorithm to see higher deviation, lowering accuracy.

Addressing these limitations will be essential to further enhance the robustness, scalability, and user experience of the proposed hand tracking method across diverse real-world applications.

## Future scope

### Proposed improvements

To address the identified limitations and enhance the performance, reliability, and scalability of the current system, several improvements are envisioned. First, enhanced vision hardware such as higher-resolution, higher-frame-rate RGB-D cameras with integrated depth sensors would improve the accuracy of ArUco marker detection, reduce latency, and mitigate visual jitter under dynamic conditions. Second, improved IMU integration using high-polling-rate inertial sensors combined with advanced sensor fusion algorithms would significantly reduce drift and improve long-term movement stability, thereby reducing the need for frequent recalibration. Third, a compact and ergonomic design is proposed, where system components are miniaturized and integrated into a single wearable unit. Such refinement would promote greater comfort and usability, especially with extended applications such as rehabilitation or immersive training. In addition, incorporation of a superior sensor suite with increased accuracy and lower latency would improve responsiveness and enable reliable tracking with quick or complex movements. Finally, marker design independence of lighting, such as with passive or IR-reflective markers, would allow reliable operation across varying light conditions and reduce the need for controlled lighting conditions. Together, such enhancements would broaden the system's employability, enabling more reliable, sustained hand tracking under real-world conditions and extending its range of application into embedded, low-power implementations.

### Potential for expanded functionality

Along with the overcoming of current limitations, the suggested system also provides promising prospects of extension of functionality and expanded applicability into greater applications. One of the significant improvements is multi-camera integration, through which synchronized networks of cameras will do away with field-of-view limitations, allowing robust hand tracking into greater or more dynamic spaces. Another direction includes application of machine learning-enabled motion prediction, which would see automatic compensation of drift, adaptable calibration, and enhanced real-time tracking accuracy through the use of predictive algorithms. Correspondingly, extension of advanced capability of gesture recognition will allow the system, through recognition of composite gestures, such as signs incorporated into sign language interpretation, AR/VR interactions, and human–robotic cooperation, provided the system has been trained with diverse and representative gesture datasets. Additionally, application-specific variations would allow deployment across applications such as medical simulation, remote robotic control, and industrial training, whereby low latency and high accuracy are critical. Lastly, modularity and reconfiguration of the system architecture would enable investigators and developers to tailor sensing configurations based upon specific application requirements, fostering reuse and reproducibility. Such extensions would make the system a scalable and flexible solution with applicability across healthcare, manufacturing, education, and immersive training, extending applicability significantly beyond the current range.

## Conclusion

This work presents a reproducible, low-cost, and modular hand tracking system that combines computer vision, capacitive touch sensing, and inertial measurement to achieve real-time gesture recognition in immersive environments. Deployed on compact hardware (Raspberry Pi Zero 2 W and ESP32), the system proves robust and responsive, even with limited computing power, facilitated by exposure tuning, region-of-interest computation, and dual-mode wireless communication via BLE and WebSocket protocols.

Comprehensive experimental analysis verifies the efficiency of the proposed approach along several aspects. The system was able to obtain 3.4 mm of localization accuracy, 85–91 % of orientation accuracy with minimal pitch and roll error, and 96.1 % of finger-state detection accuracy, together with 80–81 % of trajectory fidelity based on Procrustes analysis Latency testing further highlighted practical deployment trade-offs, with WebSocket over Wi-Fi providing higher throughput (215 KB/s, ∼467 msg/s) at the cost of increased latency (∼190 ms), while BLE offered lower latency (∼50 ms) and >98 % stability with limited throughput. Together, these results demonstrate that the system achieves a robust balance of accuracy, responsiveness, and computational efficiency, supporting its suitability for real-time VR/AR interactions.

While challenges such as yaw drift from the IMU, field-of-view constraints, and lighting sensitivity remain, these limitations can be addressed through sensor upgrades, multi-camera setups, and predictive filtering. Overall, this study contributes a practical and accessible solution to gesture-based human–computer interaction. By unifying multimodal sensing into a reproducible and experimentally validated pipeline, the system demonstrates strong potential for applications in virtual reality, education, assistive technology, and industrial automation, where reliable, low-cost, and real-time hand tracking is essential.

## Related research article

“None”.

## For a published article

“None”.

## Ethical approval

The current data set does not involve trials on humans, animal testing, or any information gathered from social media platforms.

## Funding

This research has no specific grant from funding agencies in the public, commercial, or not-for-profit sectors.

## CRediT authorship contribution statement

**Ranjeet Vasant Bidwe:** Conceptualization, Methodology, Software, Validation, Formal Analysis, Writing – Original Draft Preparation, Visualization**; Shubhangi Deokar:** Conceptualization, Methodology, Software, Validation, Formal Analysiss**; Tanisha Vyas:** Conceptualization, Methodology, Software, Validation, Formal Analysis, Investigation, Resources, Data Curation, Writing – Original Draft Preparation, Visualization**; Satviki Budhia:** Conceptualization, Methodology, Software, Validation, Formal Analysis, Investigation, Resources, Data Curation, Writing – Original Draft Preparation, Visualization; **Armaan Jeswani:** Conceptualization, Methodology, Software, Validation, Formal Analysis, Investigation, Resources, Data Curation, Writing – Original Draft Preparation, Visualization**; Yash Parkhi:** Conceptualization, Methodology, Software, Validation, Formal Analysis, Investigation, Resources, Data Curation, Writing – Original Draft Preparation, Visualization**; Nimita Jestin:** Conceptualization, Methodology, Software, Validation, Formal Analysis, Investigation, Resources, Data Curation, Writing – Original Draft Preparation, Visualization; **Utkarsh Kumar:** Conceptualization, Methodology, Software, Validation, Formal Analysis, Investigation, Resources, Data Curation, Writing – Original Draft Preparation, Visualization.s

## Declaration of competing interest

The authors declare no conflict of interest.

## Data Availability

No data was used for the research described in the article.
